# Challenges of Spatially Resolved Metabolism in Cancer Research

**DOI:** 10.3390/metabo14070383

**Published:** 2024-07-11

**Authors:** Andrew N. Lane, Richard M. Higashi, Teresa W-M. Fan

**Affiliations:** Department of Toxicology and Cancer Biology and Markey Cancer Center, University of Kentucky, 789 S. Limestone St., Lexington, KY 40536, USA; rick.higashi@uky.edu (R.M.H.); teresa.fan@uky.edu (T.W.-M.F.)

**Keywords:** stable isotope-resolved metabolomics, spatially resolved metabolism, cancer metabolism, experimental models

## Abstract

Stable isotope-resolved metabolomics comprises a critical set of technologies that can be applied to a wide variety of systems, from isolated cells to whole organisms, to define metabolic pathway usage and responses to perturbations such as drugs or mutations, as well as providing the basis for flux analysis. As the diversity of stable isotope-enriched compounds is very high, and with newer approaches to multiplexing, the coverage of metabolism is now very extensive. However, as the complexity of the model increases, including more kinds of interacting cell types and interorgan communication, the analytical complexity also increases. Further, as studies move further into spatially resolved biology, new technical problems have to be overcome owing to the small number of analytes present in the confines of a single cell or cell compartment. Here, we review the overall goals and solutions made possible by stable isotope tracing and their applications to models of increasing complexity. Finally, we discuss progress and outstanding difficulties in high-resolution spatially resolved tracer-based metabolic studies.

## 1. Introduction

### 1.1. Metabolism and Metabolomics

Metabolomics is generally described as the analytical study of the complement of metabolites in an organism or biological system (the *metabolome*), and its response to perturbations, whether intrinsic (e.g., as a result of a mutation) or extrinsic (e.g., changes in nutrient supply or other environmental factors). The identification and quantification of these molecules lie firmly in the realm of analytical chemistry. The interpretation of the analytes that are identified in terms of metabolites, as well as the underlying biochemical mechanism of their synthesis, utilization, and regulation, is the realm of metabolism.

To illustrate the distinction, all live cells take in compounds, mainly via specific membrane-bound transporters, and many are converted to other compounds by the catalytic activity of enzymes. Such compounds are part of metabolism, which comprises a network of enzymes that either capture free energy of oxidation (catabolism) or use the captured energy to drive energy-consuming reactions that build complex molecules from simpler precursors (anabolism). Metabolism also includes detoxification mechanisms involving oxidations and conjugations that are enzyme-dependent. Generally, metabolism is concerned with how these conversions are coordinated, which, in addition to identifying and quantifying the compounds that are metabolites, also requires analysis of compartmentation, protein expression, and enzyme activity via allosteric or post-translational modification. In this sense, macromolecules such as proteins, nucleic acids, complex carbohydrates, and lipids must be considered as metabolites (anabolites) as they are the products of long sequences of enzyme-catalyzed reactions. In contrast, metabolomics is, in large measure, the analytical effort of identifying and quantifying (usually) so-called small molecules, which may or may not be metabolites. There are other compounds, often small molecules, that are metabolites, but which are less commonly analyzed by metabolomics, but are clearly critical to cell function. These include oxygen, O_2_^.−^, H_2_O_2_, CO_2_, H_2_O, NO, and NH_4_^+^, none of which is organic, and all of them are both created and consumed in cells in enzyme-catalyzed reactions.

Humans rely on nutrition for many compounds in addition to those used for oxidation or anabolic purposes, including vitamins and essential amino and fatty acids, but these exogenous metabolites may be metabolized by the host. Similarly, xenobiotics may be (and often are) metabolized by the host; a wide range of enzyme activities are expressed for detoxifying foreign substances. There is also a symbiotic relationship between the host and the microbiome, which in some cases relies on the microbes to carry out one or more steps in a synthetic pathways, e.g., bile acids [1].

Even compounds that are metabolites may in fact be produced by chemical synthesis and are present in the environment. For example, lactate is the end product of lactic fermentation, and results from the reduction of pyruvate. Both enantiomers are produced biologically, though mammals produce mainly L-lactate. D-lactate can be produced by bacteria, and also from methylglyoxal [2]; these enantiomers have quite different biological properties, but are rarely differentiated by analytic tools [3]. Furthermore, lactic acid is often present in deionized water used for cell culture and is a common descaling agent that can contaminate samples. To distinguish the origin of “lactate” and determining whether it is an intrinsic or extrinsic metabolite is not always straightforward. Similarly, glutamate is in many contexts a metabolite that is involved in a large number of biochemical processes, but is also widely present in foodstuffs in the form of chemically synthesized MSG as well as a natural product. Further, especially in biofluids, compounds present at very low levels might be important potent bioactive species or just general chemical noise arising from multiple substances from air and water, pollutants, etc. Also, in the context of biofluids which distribute and disperse a very wide range of compounds by virtue of being in contact with a large surface area of cells, some fraction of which is constantly dying, a certain level of metabolites from these cells will be present in blood and urine. An abundant metabolite at 1 mM intracellular concentration in a cell volume of approx. 1 pL is 1 fmol. The content of 5 million dying cells disgorged into 5 L of blood (typical human blood volume) corresponds to a concentration of 1 nM, a level routinely measured by mass spectrometry. However, the steady-state level would be lower owing to depuration and reuptake by other cells.

The use of stable isotope tracers can certainly help with the assignment of the term “metabolite” and distinguish them from accidentally ingested adventitious compounds, which typically will not be isotopically enriched.

The critical points about enzymes are that they are catalysts (i.e., they accelerate reactions equally in both directions, but do not influence equilibrium distributions), but by virtue of high specificity, can influence flux through different branches of the metabolic network. Enzyme activity depends not only on intrinsic catalytic power but also on expression level, substrate availability, location (compartmentation), concentration of non-covalent modulators (allosteric or active site), and activity of post-translational modification enzymes (phosphorylation, glycosylation, succinate, myristylation, etc.). This is all part of the metabolic network. Many enzymes have different isoforms, which may be differentially expressed among tissues or compartments, and which may have different catalytic powers and mechanisms of activity modulation. For example, the enzyme PFK1 catalyzes the phosphorylation of fructose-6-P (F6P) to fructose 1,6 bisphosphate coupled to the conversion of ATP to ADP. This enzyme exists as three isoforms in mammals, the muscle, liver, and platelet isoforms [4], and mainly resides in the cytoplasm, though it is also found in the nucleus [5,6]. PFK1 is also allosterically inhibited by ATP (i.e., there is a separate ATP binding site), but is allosterically activated by AMP. It may also be inhibited by high concentrations of citrate and PEP. Potentially the most potent allosteric activator of PFK1 is fructose 2,6 bisphosphate, which is synthesized from F6P via the activity of one of the four isoforms of the unrelated bienzyme complexes of PFFKB [7]. These enzymes contain both PFK2 activity and the specific F2,6 phosphatase activity, and the relative levels of these competing functions depend on both isoforms and the level of phosphorylation by PFKB kinase [7,8]. Furthermore, PFK1 may undergo O-GlcNAcylation, especially under hypoxia, which decreases PFK1 activity [9].

**Figure 1:**
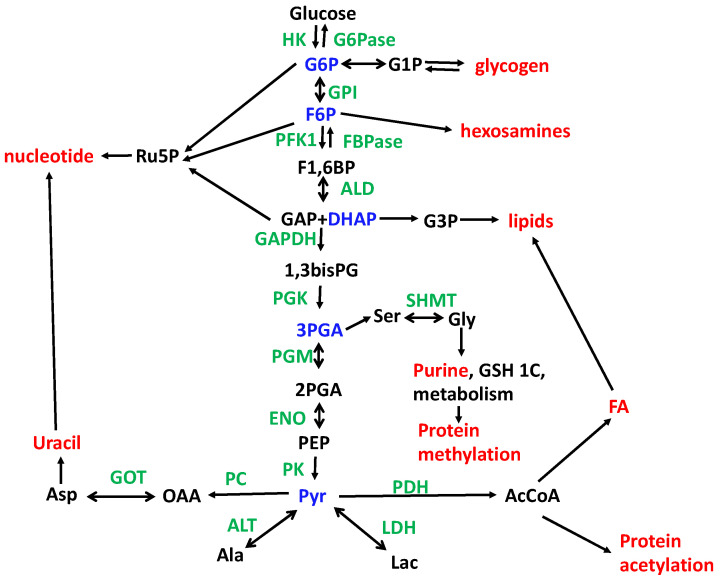
**Glycolysis as a source of anabolic intermediates.** PFK1 occupies a crossroad that determines relative flux into glycolysis versus the oxidative branch (NADPH generating and Rib-5-P) of the pentose phosphate pathway. Double-headed arrows represent reversible reactions, catalyzed by the same enzyme; two arrows represent reactions catalyzed by different enzymes. Glycolytic anabolic precursors are in blue; anabolic products are in red; enzyme names are in green. G6P: glucose-6-phosphate; F6P: fructose-6-phosphate; F1,6BP: fructose-1,6-bisphosphate; GAP: glyceraldehyde-3-phosphate; DHAP: dihydroxyacetone phosphate; 1,3bisPG: 1,3-bisphosphoglycerate; 2PGA: 2-phosphogycerate; PEP: phosphoenolpyruvate; Pyr: pyruvate; OAA: oxaloacetate; AcCoA: acetyl CoA; Lac: lactate; Ru5P: ribose-5-phosphate; GSH: reduced glutathione; HK: hexokinase; G6Pase: glucose-6-phosphatase; PGI: phosphoglucose isomerase; PFK1: phosphofructokinase 1; FBPase: fructose 1,6bisphosphatase; ALD: aldolase; GAPDH: glyceraldehyde-3-phosphate dehydrogenase; PGK: phosphoglycerate kinase; PGM: phosphoglycerate mutase; ENO: enolase; PK: pyruvate kinase; LDH: lactate dehydrogenase; ALT: alanine aminotransferase; PC: pyruvate carboxylase; PDH: pyruvate dehydrogenase; SHMT: serine hydroxymethyl transferase; GOT: glutamate oxaloacetate aminotransferase.

PFK1 occupies a crossroad that determines relative flux into glycolysis versus the oxidative branch (NADPH generating and Rib-5-P) of the pentose phosphate pathway (Figure 1), and is strongly linked to metabolic energy levels (ATP) via glycolysis, as well as the provision of precursors for lipid synthesis (via DHAP), glycosylation (via F6P and the hexosamine pathway), and serine/glycogen production (via 3-phosphoglycerate).

Mapping metabolic pathways within complex networks is best achieved using tracers, which is typically achieved using stable isotope-enriched precursors with mass spectrometry and NMR as the isotope-sensitive analytical tools [10,11,12,13,14], though it is also possible to use vibrational spectroscopy under certain circumstances [15,16]. NMR identifies molecules and isotopomers of a subset of abundant metabolites, and quantification of individual isotopomers can be achieved using a variety of NMR methods including selection by isotope editing [12,17]. Mass spectrometry methods generally have a higher metabolic coverage and, combined with chromatography and fragmentation, can identify and quantify a large number of compounds and their isotopologues (number of heavy atoms incorporated). With Tandem MS, it is possible to determine which metabolic subunits contain the exogenously supplied label, for example, in the ribose and nucleobase subunits of nucleotides and dinucleotides [18,19] and intact complex lipids [20]. With ultra-high-resolution mass spectrometry (>20,000 at *m*/*z* = 200, [10,21]), it is possible to discriminate the incorporation of a single ^13^C, a single ^15^N, or a single ^2^H atom (all nominally +1 amu) into a product, allowing multiplexed tracing to be carried out [19,21]. For mass spectrometry detection, the natural abundance must also be taken into account, as for large molecules, the m + 1 ion can be quite substantial. Standard methods are available for this kind of correction for both single isotopes [22] and multiple isotopes [23].

Although time courses of metabolite concentrations (or amounts) can provide valuable kinetic information, especially about nutrient uptake rates and excretion [24,25,26], the determination of metabolic fluxes more generally requires a metabolic model that can be parametrized, for either dynamic (time-dependent) or isotopic steady-state conditions [19,26,27,28,29,30,31,32,33]. Stable isotope tracing is a powerful means to determine the metabolic network activity in the system of interest, but generally, is not flux per se [30].

### 1.2. Models and Complexity

Although it is possible to apply tracer methodologies to whole organisms, including human subjects [34,35,36,37,38,39,40,41,42], the complexity and limited experimental control can limit the amount of mechanistic information that can be recovered [40,41,42,43]. For this reason, various model systems that can be manipulated are needed. Different experimental models vary in their complexity from cells in 2D culture, 3D spheroids and organoids, excised tissues, perfused organs to whole mouse models, with or without xenografts, or genetically engineered animals [43]. Even organotypic tissue cultures, while allowing extensive control of experimental variables, by virtue of their heterogeneity in cell types and interactions, are highly complex, and some level of spatial resolution is needed [44].

In this article, we discuss progress in isotope tracing methodologies for pathway analysis, the pitfalls in modeling systems of increasing complexity [43], and the challenges in applying these approaches at single cell resolution in live tissue [15], which is becoming increasingly urgent as other omics technologies are able to measure RNA and protein expression at the subcellular level expression [45,46,47,48,49]. We will illustrate principles mainly from the perspective of cancer metabolism, immune cell metabolism, and the tumor microenvironment. Because the field is very large and is rapidly developing, we can only provide representative examples, but we have cited other reviews wherever possible.

## 2. Pathway Mapping via Tracers

Actual pathway mapping is complex and relies on identification and quantification of many metabolites and their specific isotope distributions, which can be mapped onto known pathways using standard biochemical pathways relevant to the organism and tissue type of interest found in various databases [50,51,52,53,54], and is substantially aided by expression data, especially of proteins [55]. In the following, we review stable isotope tracing of several intersecting metabolic pathways.

### 2.1. Tracers for Metabolism

If metabolic pathways were completely independent of one another, pathway mapping and utilization would be simple, and measuring the end product as a function of time would determine the pathway utilization. In practice, metabolic pathways are components of a complex intersecting network (Figure 1). For example, the conversion of glucose to pyruvate (glycolysis) has numerous branch points where carbon is removed from the pathway (i.e., at G6P, F6P, DHAP, 3PGA, pyruvate). Indeed, the end product pyruvate has numerous fates: lactate (via lactate hydrogenase), alanine (via glutamate pyruvate transaminase), AcCoA (via pyruvate dehydrogenase), and oxalacetate (via pyruvate carboxylase). Thus, glucose uptake and glycolysis are intimately connected to glycogen metabolism, the pentose phosphate pathway to generate ribose-5-phosphate and (in the oxidative branch) NADPH, the hexosamine pathway, glycerol and complex lipid biosynthesis, serine and 1-carbon metabolism, the Krebs cycle, anaplerosis, and both pyrimidine and glutathione biosynthesis. Glucose is not the only carbohydrate that can feed these pathways; other common dietary carbohydrates such as fructose and galactose can be utilized. Galactose via the Leloir pathway has been reported to be important in glioblastoma multiforme [56] and hepatocellular carcinoma [57], and fructose is associated with liver and lipid metabolism [58,59,60]. However, compared with glucose utilization, their contributions to cancer metabolism have not been studied in great detail [61].

**Figure 2:**
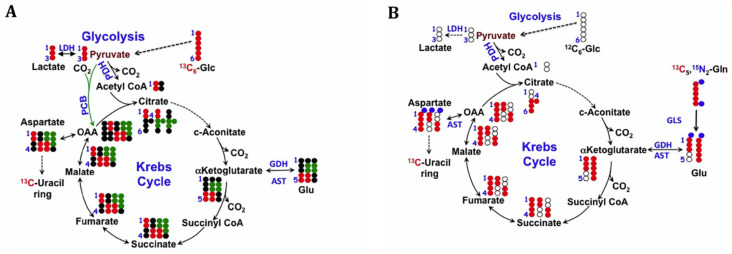
**Isotopomer evolution in the Krebs Cycle**. Acetyl CoA produced by oxidation of [U-^13^C]-glucose-derived pyruvate (PDH) may enter the Krebs cycle and label intermediates 2 carbon atoms at a time. Acetyl CoA may also be exported from the mitochondria as citrate, when ATP citrate lyase converts the cytoplasmic citrate to OAA + AcCoA, which is used for fatty acid synthesis and acetylation reactions. Anaplerosis from Gln via glutaminase and transaminases and/or from pyruvate carboxylation also contributes carbon to the TCA cycle intermediates, leading to complex isotopomer patterns as increasing numbers of cycles are traversed. (**A**) ^13^C_6_ glucose plus unlabeled Gln. Black dots are ^12^C. ^13^C_6_ glucose (red dots) produces ^13^C_3_ pyruvate which can enter the Krebs cycle either via PDH as a 2-carbon unit via acetyl CoA or a 3-carbon unit via PCB (green dots), which give rise to different isotopomers of Krebs cycle intermediates and anabolic products such as uracil and its precursor Asp, which are distinguishable by NMR as ^13^C-1,2 and ^13^C-3,4 Asp via PDH in the forward direction, and ^13^C1,2,3 Asp via PCB. (**B**) ^13^C_5_,^15^N_2_ Gln plus unlabeled glucose. Open black circles are ^12^C. Red dots are ^13^C atoms from Gln; blue dots are the amino and amido nitrogen atoms of Gln. The anaplerotic input of fully labeled Gln produces the fully labeled Krebs cycle intermediates starting from 2OG via glutaminase and GDH or aminotransferases. Fully labeled OAA from Gln condenses with glucose-derived AcCoA to produce quadruple-labeled citrate, which becomes triply labeled 2OG via the Krebs cycle and doubly labeled succinate. AST will transaminate OAA with Glu, transferring the amino nitrogen to Asp. Unlabeled glucose produces the partially labeled 2OG and subsequent metabolites with scrambling at the succinate-to-fumarate step. The isotopomers produced evolve in further cycles and differ with other inputs to the cycle. Detailed isotopomer analysis is needed to disentangle the effects such as with TCAsim [29]. LDH: lactate dehydrogenase; PDH: pyruvate dehydrogenase; PCB: pyruvate carboxylase; GLS: glutaminase; GDH: glutamate dehydrogenase; AST: aspartate aminotransferase (also glutamate-oxoglutarate aminotransferase, GOT). From Figure 2 of Lane and Fan (2017) [65].

Acetyl CoA produced by oxidation of pyruvate (PDH) may enter the Krebs cycle and label intermediates two carbon atoms at a time (Figure 2). Acetyl CoA may also be exported as citrate from the mitochondria to the cytoplasm, where ATP citrate lyase converts the cytoplasmic citrate to AcCoA + OAA. The AcCoA produced may then be used for fatty acid (FA) biosynthesis. The OAA may be reduced by cytoplasmic MDH and participate in the redox shuttle mechanism for re-oxidizing cytoplasmic NADH to NAD^+^. The shuttle mechanism is essential for oxidizing cytoplasmic NADH that is not re-oxidized in the LDH-catalyzed reaction. Figure 2 shows how Krebs cycle intermediates are labeled from [U-^13^C]-glucose via both PDH and PC reactions. Careful quantitative analysis of isotopologues of malate, citrate, and glutamate can determine the relative input from PC and PDH [34,62]. [^13^C1,2]-glucose labeling has also been used for analyzing the PC versus PDH input to the Krebs cycle [63,64].

Parsing the substrate participation from glucose and amino acids such as glutamine requires tracers [66]. There are numerous commercially available forms of stable isotope-enriched precursors that can be chosen according to the experimental question being addressed. For example, several ^13^C isotopomers of glucose are available, including [U-^13^C]-glucose, ^13^C-1, ^13^C-2, ^13^C1-,2, and ^13^C-3,4 as well as deuterated forms, e.g., ^2^H_7_ and ^2^H6,6 glucose. Similarly, [U-^13^C], [U-^13^C,^15^N]-, ^15^N_2_, ^13^C_1_ or ^13^C_5_ Gln can be used to probe different aspects of Gln metabolism. Other amino acids labeled with ^13^C, ^15^N, or ^2^H can also be obtained, as can ^13^C or ^2^H FA and ^13^C fructose. Many other enriched compounds are commercially available for specific questions. Table 1 summarizes the parts of metabolic networks that these tracers probe.

For example, [U-^13^C]-glucose is valuable for tracing central carbohydrate metabolism. Because of the abundant glucose transporters, most cells readily absorb glucose, which is immediately phosphorylated to G6P, and trapped. The fate of the G6P then depends on many conditions and is the precursor to glycogen synthesis (via UDP-glucose) and the pentose phosphate pathway or is committed to glycolysis (Figure 1). Most of the intermediates of glycolysis and the pentose phosphate pathway can be identified and quantified by IC-FTMS [17,18,98].

### 2.2. Glycolysis and Lactic Fermentation

G6P is oxidized by NAD^+^ to pyruvate in glycolysis, generating NADH and ATP. The pools of the intermediates are typically rapidly replaced (minutes) with uniformly ^13^C labeled versions at high fractional enrichment. In cancer cells in culture, the levels of free glucose and G6P are typically low, indicating that, at the concentrations of exogenous glucose typically supplied (5–25 mM), uptake and phosphorylation (Glut + HK activity) are relatively low compared with the activity of downstream steps (PFK through PK). Indeed, in cancer cells and tissues, the flux control coefficients for glycolysis are usually highest for Glut and HK, rather than the often described “rate limiting steps” of PFK1 and PK activities [99,100,101,102,103], which may be more important in normal tissue. This is likely to be exacerbated in solid tumors that are poorly vascularized and where nutrient delivery is compromised (see below). PFKB4 produces fructose-2,6 bisphosphate, a potent activator of PFK1, and decreases its glycolytic flux control coefficient, and this may also be important for net flux control in glycolysis [101] versus diversion to other pathways (cf. Figure 1). Note that cell culture media are typically highly unphysiological. Even in serum, the glucose concentration is 4–6 mM (and considerably lower in poorly vascularized tissue), and amino acid concentrations greatly exceed those of serum. More recently, media formulations that match those of human serum are being made available [104], which can significantly impact the biochemical behavior of cultured cells [104,105,106].

Pyruvate, the end product of glycolysis, has numerous fates. In cancer cells, much of the glycolytically generated pyruvate is converted to lactate via LDH and excreted along with a glycolytically produced proton, regenerating NAD^+^ to allow glycolysis to continue, and also helping maintain a near-neutral intracellular pH [107]. This lactic fermentation can account for most of the glucose consumed by the cell [71,85,108]. However, some pyruvate may be transported into and oxidized by the mitochondria via the Krebs cycle, which also requires functional respiration. Mitochondrial pyruvate may also be carboxylated to OAA by PC, an anaplerotic reaction that is important in some cancers such as that of the lung [34,37,62]. Cytoplasmic pyruvate may also be transaminated to alanine (by ALT), some of which is excreted [62], and some enters protein synthesis or simply buffers the free pyruvate.

As Figure 1 shows, not all of the exogenous glucose that is consumed completes glycolysis, and in addition, glycogen hydrolysis may also contribute to carbon flow through the glycolytic pathway. Significant amounts of glucose carbon may be consumed in any of several branch points of glycolysis. G6P can be oxidized to ribose-5-P +CO_2_ in the oxidative branch, generating 2 moles of NADPH. The resulting pentose phosphate is a precursor for nucleotide biosynthesis, which is very important in dividing cells, but may also be returned to glycolysis through the reversible steps of the non-oxidative branch of the pentose phosphate pathway (PPP). In red blood corpuscles, this is the major fate of glucose-derived pentose phosphate carbon [109]. The labeling patterns of the PPP can become highly complex via the transaldolase (TA) and transketolase (TK) reactions, and the relative importance of the oxidative and non-oxidative pathways cannot be easily discriminated using^13^C_6_ glucose. ^13^C1,2 or ^13^C1,6 glucose, however, can discriminate, because C-1 of glucose is lost in the PPPox but is retained in the non-oxidative branch [110]. IC-FTMS can determine most of the PPP intermediates, and NMR is particularly useful for isotopomer analysis of the ribose in newly synthesized nucleotides [110] and, ultimately, in RNA [111].

Although the PPP may account for only a few percent of glucose consumption, other branches from glycolysis may also divert carbon from pyruvate production. Fructose-6-phosphate (F6P) is the precursor to UDP-GlcNAc synthesis, which is used for specific protein glycosylation, both O- and N-linked. In dividing cells, where the total protein content is doubled in each cell division, de novo glycosylation must occur, which may account for a significant amount of consumed glucose [112], though specific determinations using tracers have not been widely reported.

Dividing cells also make complex lipids which are dominated by glycerophospholipids and triglycerides. The glycerol backbone derives mainly from glucose via DHAP, as is readily shown in lipid analyses by NMR and MS of cells treated with [U-^13^C]-glucose. Typically, the glycerol backbone is triply labeled when ^13^C_6_ glucose is the source, with very little scrambling. Exogenous glycerol may also end up in complex lipids [110,113]. The fatty acyl chains are synthesized from AcCoA, deriving from citrate. Depending on the cell type and growth conditions, a significant portion of the fatty acyl chain carbon may also be derived from glucose, with contributions from glutamine and exogenous free fatty acids [59,88,110,114,115,116,117,118,119,120,121].

A fourth significant branch point in glycolysis occurs at 3-phosphoglycerate. PHGDH catalyzes the oxidation of 3PGA as the entry point into the synthesis of serine and then glycine [19,122,123,124,125]. Both amino acids may also be taken up from the medium. Serine and glycine are used for protein synthesis, but also for phospholipid synthesis (cf. phosphatidyl serine and sphingolipids), purine biosynthesis, and 1-carbon metabolism (glycine). The relative importance of the uptake of exogenous amino acids versus synthesis from glucose is highly cell- and condition-dependent and requires multiple tracers to determine the fate of different atoms into products.

Therefore, the amount of glucose carbon consumed will generally be higher than the amount of glucose-derived pyruvate produced. The actual fraction of glucose consumed that enters these anabolic pathways is variable and requires careful quantification (see above).

As pyruvate is also generated from other reactions (e.g., via malic enzyme) and has multiple fates, steady-state levels of either pyruvate or the commonly proposed surrogate lactate, even using ^13^C glucose and monitoring ^13^C lactate, can only provide an upper and lower limit to the total glycolysis. However, a useful parameter is to determine how much ^13^C glucose was consumed per cell and how much of that glucose was converted to ^13^C lactate or ^13^C Ala and excreted into the medium at a certain time (Equation (1)).

F(t) = 0.5*Δ [Lac(t)]/Δ[Glc(t)](1)

For example, in a 2D cell culture using a 10 cm plate, the medium volume would be 8 mL, and for 1 million epithelial cells of volume 2 pL, the cell volume would be 2 μL (volume ratio = 4000:1), of which approx. 30% is biomass (<0.5 pg/cell). For an initial cell number of 1 million, after one doubling time, the amount of new cellular biomass would be of the order 50 ng. At most, 50% of this biomass could be glucose-derived (protein is the most abundant component of cellular biomass, and nearly half of the amino acids are essential, i.e., cannot be made from glucose [126,127]). This amounts to <1.5 nmol of glucose being incorporated into new cellular biomass. For a medium glucose concentration of 10 mM, the amount of glucose initially present in the medium = 80 μmol; even a small consumption over a single cell doubling (ca. 24 h) very greatly exceeds the amount of biomass produced. In fact, cancer cells typically consume glucose at a high rate, easily 40 μmol over a period of one cell doubling from 1 million cells. Therefore, most of the glucose that is consumed does not enter biomass, but is either oxidized to CO_2_ and lost, converted to excreted metabolites such as lactate, alanine, glutamate, and acetate [82], or produces extracellular matrix including collagens, other proteins, and possibly polymeric carbohydrates [128,129,130,131]. In fact, all of these metabolic processes occur, to different extents, according to cell types and growth conditions [71,85,122,132]. Lactate production and excretion is often a major end product of glucose metabolism in proliferating cells [133], though in tumors, it can also be a fuel [35], but does not always account for the majority of the glucose consumed. Ala, acetate, and Glu are biologically significant excretion products, but usually account for much less carbon than lactate. CO_2_ production is potentially a major end product of glucose oxidation, primarily via mitochondrial oxidative decarboxylation, but there are few studies of the mass balance between glucose consumption and CO_2_ production, not least because cell culture is usually carried out in media containing 12–25 mM bicarbonate and a 5% CO_2_ atmosphere (and at 20% oxygen), which might serve to suppress oxidation in favor of fixation (cf. mitochondrial reductive carboxylation [71,118,119,120,121]). More realistic tissue conditions may have alternative explanations [134]. Although it is often assumed that fibroblasts provide the extracellular matrix (implying a rather large amount of metabolism devoted to producing the polymeric materials, not only in terms of carbon, nitrogen, and oxygen, but also the metabolic energy to drive anabolism), other cells, including cancers, produce ECM components, especially collagens [129]. Matrix protein deposition in 2D or 3D culture can potentially interfere with metabolite normalizations if total protein is used, depending on the method of cell harvesting. Moreover, the metabolic requirements of matrix deposition in tissue or cell culture have not been quantitatively evaluated.

#### Glycolytic Rate

The glycolytic rate, or flux, is the rate at which glucose is oxidized to pyruvate. As alluded to above, the measurement of this flux is not straightforward. As glycolysis produces protons, the rate of extracellular acidification (ECAR) is proportional to the glycolytic flux [135]. However, the degree of acidification as measured by proton activity (pH) depends on buffer capacity, as well as any other proton-producing reactions [136]. More recently, deuterium NMR has been used to estimate glycolytic flux in vivo, using deuterated glucose and measuring the deuterium released via glycolysis into water [75,135,136,137].

### 2.3. Gluconeogenesis

Gluconeogenesis is the production of glucose from precursors downstream of pyruvate in the metabolic network, and requires four alternative enzyme-catalyzed reactions to those of glycolysis, namely PC and PEPCK, which catalyze the conversion of pyruvate to PEP via OAA; F1,6 bisphosphatase, which converts FBP to F6P; and G6P phosphatase, which concerts G6P to glucose, which can be readily exported from the cell via glucose transporters. Usually, the full gluconeogenic pathway is active in the liver and kidneys [138], which helps maintain systemic glucose homeostasis by converting (mainly) lactate and alanine to glucose, though other glucogenic amino acids can also be used, at the cost of metabolic energy [138,139,140,141,142,143]. However, in the tumor microenvironment, glucose is often highly depleted, leading to low concentrations [144,145,146], which contributes to the flux control at the transporter level (see above). As glycolysis provides several anabolic precursors required for cell proliferation (see above), low glucose availability can impact a cell’s survival and ability to divide [147,148]. Further, cancer cells can express PC [34,149] and PEPCK (PCK) [150,151] and suppress FBPase [152] and also take up lactate as fuel [35,62,153]. Thus, lactate, Ala, and other glucogenic amino acids can provide carbon for the anabolic precursors which require bypassing the PK-catalyzed step to trioses like 3PGA (serine/glycine/1-C pathway needed for purine biosynthesis) and G3P (lipid biosynthesis) as well as the PPP (F6P + GAP), HBP (F6P), and if FBPase is active, also G6P for the oxidative branch of the PPP (Figure 1).

### 2.4. Redox Metabolism

The redox couples NAD^+^/NADH, NADP^+^/NAPH, and FAD/FADH2 differ depending on the cellular compartment and reflect the different catabolic and anabolic needs in these compartments [154]. Thus, the free [NAD^+^] to [NADH] ratio is very high in the cytoplasm of most cells [155], and significantly lower in the mitochondrial matrix [156]. In contrast, the NADP^+^ to NADPH ratio is <<1 in the cytoplasm [157], and higher inside mitochondria [158,159]. The ratios also depend on conditions of substrate availability and oxygenation [160]. As indicated above, a high cytoplasmic NAD^+^/NADH ratio is needed to maintain a high glycolytic flux, whereas a high cytoplasmic NADPH/NADP^+^ ratio is needed to maintain fatty acid synthesis and the reduction of ribonucleotides for DNA synthesis in S-phase, as well as to combat oxidative stress via glutathione reductase [158,161]. An important set of experiments using stable isotope tracers with deuterated substrates was used to show the activity dehydrogenases as they accepted a deuteron onto NADP^+^ to form NADPD [159,162]. In these experiments, the substrates were 3 or 4-^2^H glucose to measure NADPH production from the oxidative PPP, or 2,3,3,4,4-^2^H-glutamine and 2,3,3-^2^H-aspartate, which implicated activity of GDH and malic enzyme, and 2,3,3-^2^H-serine, which implicated the activity of serine hydroxymethyltransferases (SHMTs) and methylene tetrahydrofolate dehydrogenases. Discrimination between cytosolic and mitochondrial isoforms, however, has to conformed by knockout experiments. In principle, the same approach could be used for flavin proteins using, for example, deuterated succinate (provided as dimethylsuccinate, for example), or other specific dehydrogenases where the deuterated substrate can be introduced into cells.

Compartmented metabolism may also be deconvoluted using a combination of tracers with specific modeling [19,163]. It is also possible to parse compartmented metabolism by isolating organelles from cells, such as mitochondria [164] and nuclei [6].

**Figure 3:**
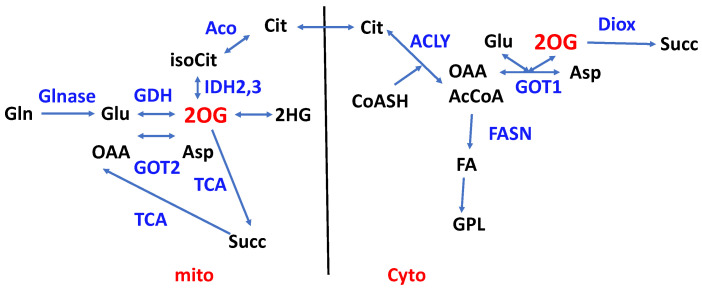
**2OG is a hub metabolite with several fates in both the mitochondria and the cytoplasm**. Mitochondrial 2OG may derive from IDH activity in the TCA (Aco + IDH2,3), which is reversible depending on substrate and redox ratios; interconversion to 2HG via specific dehydrogenases or variant IDH2; transamination of Glu via GOT2, from Gln via glutaminase; and oxidization to citrate and then OAA via the TCA. In the cytoplasm, citrate is cleaved to OAA + AcCoA, which itself has numerous fates including fatty acid synthesis; OAA may transaminate with Glu (via GOT1) producing 2OG, which is a co-substrate for 2OG-dependent dioxygenase (Diox). Enzymes in blue: Glnase glutaminase; GDH: glutamate dehydrogenase; GOT2: glutamate OAA transaminase 2; IDH2: NADP-dependent isocitrate dehydrogenase; IDH3: NAD-dependent isocitrate dehydrogenase; TCA: tricarboxylic acid cycle; Aco: aconitase; ACLY: ATP-dependent citrate lyase; FASN: fatty acid synthase; GOT1: glutamate OAA transaminase 1; Diox: 2OG-dependent dioxygenase (numerous).

The GSH/GSSG ratio is also a redox couple, and although the two forms are in continuous exchange, GSH also can modify proteins and is diluted by cell division, necessitating continuous synthesis to maintain homeostasis. GSH is a tripeptide of Gly, Glu, and Cys, so its synthesis necessitates the uptake and/or synthesis of these three amino acids. Notably, Glu is the transamination product of 2OG (Figure 2 and Figure 3), Cystine is antiported with Glu via the Xc^−^ system [165], and Gly is produced from serine. The synthesis of GSH is therefore closely linked to central, amino acid, and redox metabolism, and its synthesis and turnover are fundamental to cell survival and proliferation. Tracer methodology that can measure several pathways simultaneously is therefore needed to determine GSH/GSSG homeostasis.

#### 2.4.1. Reductive Carboxylation of 2OG

2OG has several fates in cells (Figure 3), both as a TCA cycle intermediate and as a co-substrate for 2OG-dependent dioxygenases [166]. In mitochondria, 2OG is produced by the reversible activity of the unrelated NADP^+^-dependent IDH2 and NAD^+^-dependent IDH3. It is also produced by successive deamidation by glutaminase and amidotransferases to glutamate followed by transamination with partner amino acids or oxidative deamination via GDH activity (NAD(P)-dependent). 2OG may also be produced in the cytoplasm by the activity of IDH1 [167] (Figure 3).

The IDH-catalyzed reaction is described by

Isocitrate + NAD(P)^+^ ⇔ 2OG + NAD(P)H + CO_2_ (2A)

The equilibrium constant for this reaction is ca. 1 M [168], and the preferred substrate for carboxylation is dissolved CO_2_ rather than bicarbonate [169]. The net direction of flux is determined by the actual concentrations of the substrates and products, such that if

[2OG][NAD(P)H][CO_2_]/[isocitrate][NAD(P)^+^] > K_eq_ (2B)

then the net reaction is toward isocitrate synthesis, i.e., reductive carboxylation.

Reductive carboxylation from Gln-derived 2OG via IDH1 or IDH2 has been observed in brown adipose tissue [170] and in cancer cells under deep hypoxia or with defective mitochondria [71,88,108,118,120]. Defining reductive carboxylation unambiguously requires a careful choice of which tracers to use and which products to measure (Table 1), preferably coupled with manipulation of critical enzyme activities [108,171].

Under deep hypoxia, or where mitochondria have a diminished ability to respire, the NADPH/NADP^+^ level tends to rise. Furthermore, in standard cell culture, the concentrations of CO_2_ and bicarbonate are also very high, all of which favor reductive carboxylation. Indeed, as the reaction is reversible, tracers such as ^13^C from Gln will exchange even if the net flux is toward decarboxylation [134], which should be accounted for in all tracer experiments.

The disequilibrium ratio of 2OG to isocitrate depends on reactions that consume or produce these compounds and the NADP^+^/NADPH redox ratio. Critically, whether mitochondrial or cytoplasmic, aconitase equilibrates isocitrate with citrate, and then ATP-dependent citrate lyase converts the citrate to AcCoA and OAA, driven by the hydrolysis of ATP. Subsequent consumption of AcCoA to make FA is further driven by NADPH oxidation and ATP hydrolysis (Figure 3); overall, this can be a highly favorable process, but the fluxes depend on the actual enzyme activities and the various metabolite concentration ratios, which depend heavily on the growth conditions. Nevertheless, hypoxia and/or defective mitochondria seem to be favored in cultured cancer cells, where de novo FA synthesis under normoxia is typically low [88,110,116] and may compete with exogenous FA uptake [95,97,172], especially in vivo [173,174].

Interestingly, the reaction catalyzed by malic enzyme has an equilibrium constant of ≈30 M^−1^ for [malate][NADP^+^]/[pyruvate][NADPH][CO_2_] [175], indicating a reversible reaction that will be associated with a significant exchange flux. The net flux is likely to be poised [175], as well as being linked to CO_2_ hydration and thus carbonic hydratase activity, which should be taken into consideration in tracer experiments [168].

2-hydroxyglutarate (2HG) (Figure 3) is a naturally occurring metabolite that occurs in both enantiomeric forms, normally from the reaction catalyzed by D and L-2OG dehydrogenases [176,177,178], which if absent or inactive cause 2HG acidurias [179]. 2HG is normally present at low levels but may increase enormously when there are specific mutations in the genes encoding IDH1 or IDH2, and which are associated with several cancers including gliomas [167,180], leukemias [181,182], and renal cell carcinomas [183]. 2HG is a potent inhibitor of 2OG-dependent dioxygenases [184,185], and therefore may have a range of downstream biochemical effects [184]. The variant IDH enzymes use NADPH to reduce 2OG to 2HG, typically producing the D enantiomer [3,186] and potentially skewing the NADP^+^:NADPH ratio [187]. Other enzymes including MDH, LDH, and PHGDH may produce alternative enantiomers [185,188]. As the two enantiomers may have different biological effects, the determination of the enantiomeric purity can become important. As neither MS nor NMR can directly discriminate enantiomers, it is necessary either to use a chiral column or to convert the enantiomers to distinguishable diastereomers using a chiral derivatizing agent [3,188,189], or via a chiral shift reagent [190]. There are other metabolites that exist biologically as enantiomeric pairs, which are almost always analyzed in total. Although protein amino acids are almost always the L form, D amino acids are produced naturally and have quite different biological effects to the L form [185]. Mammalian cells produce almost exclusively L-lactate, though some D-lactate may be produced by the methylglyoxal pathway especially under conditions of high glycolytic flux [191], but some bacteria produce the D enantiomer [192]. As a 2-hydroxy carboxylic acid, D- and L-lactate can also be discriminated by the same chiral derivatization of 2HG [3].

#### 2.4.2. Dinucleotide Metabolism

NAD(P)^+^ and their reduced forms are critical to central redox metabolism, and the total amount of these dinucleotides is strongly regulated. Dividing cells and cells that have active ADP ribosylation must maintain their overall NAD^+^ levels. NAD^+^ synthesis uses either the de novo pathway or salvage pathways [193]. The latter can only maintain levels, whereas in proliferating cells, net NAD^+^ synthesis is required. NAD^+^ is synthesized from ATP and nicotinamide riboside. De novo synthesis of ATP utilizes the PPP and purine pathways as described in Section 2.5.2. Nicotinamide riboside is synthesized from nicotinamide and PRPP (from the pentose phosphate pathway) by the agency of Nicotinamide phosphoribosyltransferase (NAMPT) or in the de novo kynurenine pathway from tryptophan from quinolinate via quinolinate phosphoribosyltransferase (QPRT) [194]. The nicotinamide derives either from exogenous sources (niacin, or vitamin B_3_), from NAD^+^ turnover via ADP ribosylation, or from de novo synthesis from tryptophan via the kynurenine pathway [194,195]. Nicotinamide itself can also be methylated to 1Me-Nicotinamide, which may be either retained by the cell and impact SAM levels and thence 1-carbon metabolism [196,197,198] or excreted as an immune suppressive product [199,200]. The kynurenine pathway is particularly expressed in the liver, which can supply systemic nicotinamide under conditions of vitamin B_3_ deficiency [91]. However, other tissues also express the enzymes, as well as, notably, in some cancers [90], where one of the early products, kynurenine, is an immune suppressor in the TME [201].

Tryptophan metabolism through the kynurenine pathway can be traced in individual cells or in animal models using stable isotope tracing. Fan et al. [90] used ^15^N_2_ tryptophan to determine the pathway in human macrophages, and Liu et al. [91] used ^2^H_4_ NAM or [U-^13^C] tryptophan to follow the uptake and metabolism of these precursors into NAD in breast cancer cells and mice. As expected, in the cells, exogenous NAM accounts for most of the newly synthesized NAD^+^. In mice, [U-^13^C] tryptophan was converted to NAD^+^ primarily in the liver with some kidney labeling, but only low levels in other tissues, which showed much higher levels of ^2^H-labeled NAM and NAD^+^.

### 2.5. Macromolecule Metabolism

Macromolecules are anabolites synthesized from small metabolite subunits with the expenditure of metabolic energy. Proteins comprise the most abundant class of macromolecules in cells. For an average protein concentration of 20%, w/volume, the concentration of peptides is 1.8–2 M, or ca. 2–4 pmol/cell. One cell doubling therefore requires a large amount of amino acids, either taken up from the exterior or synthesized de novo, and each peptide bond uses ca. 5 ATP equivalents [202,203]. Protein synthesis is the most energy-expensive process in the cell. Further, protein degradation via the proteasome also uses ATP hydrolysis. Approximately half of the protein amino acids are essential and therefore must be transported from outside, whereas the other amino acids can be synthesized from other metabolites or may also be transported from outside the cell. Some amino acids are energetically neutral to synthesis (e.g., Ala from Pyr and Glu by transamination), whereas others also cost metabolic energy (e.g., glutamine and asparagine synthesis). Although the NMR and X-ray crystallography communities have long used metabolic incorporation of stable isotopes into proteins, this is aimed at high or defined levels of incorporation for structural and dynamic analysis of purified proteins [204,205,206,207]. To assess protein synthesis and turnover, the traditional method uses radioisotope labels, such as ^35^S-Methionine in pulse chase experiments, which have numerous disadvantages [208].

#### 2.5.1. Protein Analysis

Determining protein synthesis and/or degradation requires tracers. Making links to central and amino acid metabolism needs amino acid analysis that is tracer-sensitive. Although there are many methods for protein analysis, they are often not compatible with isotope enrichment.

The traditional method of protein hydrolysis using 6 N HCl is time-consuming and destroys several amino acids (e.g., tryptophan, methionine) or converts them to other amino acids (Gln -> Glu, Asn -> Asp) [209], resulting in loss of information. Although a cocktail of proteases can also hydrolyze proteins under mild conditions [210], this is slow and may contaminate the sample with unlabeled amino acids. Alternatively, rapid acid hydrolysis using focused microwaves achieves complete conversion in only 10 min, with retention of the labile amino acids [21,211]. The subsequently derivatized amino acids with ethyl chloroformate could be analyzed completely by direct-infusion ultra-high-resolution mass spectrometry on cell and tissue samples simultaneously treated with multiple tracers [21]. Under these conditions, only non-essential amino acids are labeled in mammals, though with careful choice of the labeling scheme, it is possible to discriminate between de novo synthesis versus uptake of exogenous amino acids that are incorporated into proteins. For essential amino acids, of course, these can be provided as ^13^C- or ^15^N- or ^2^H-enriched compounds, and then their uptake and incorporation into protein can be measured [92,212]. The protein pool may be a major sink of exogenous glucose where non-essential amino acid production is significant.

#### 2.5.2. Nucleic Acid Analysis

**Figure 4:**
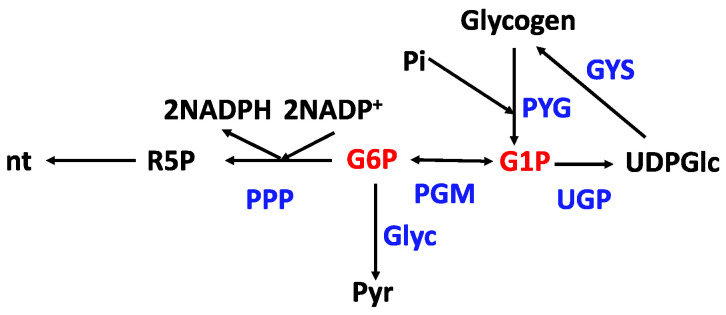
**G6P/G1P is a node for glycolysis, glycogen metabolism, and the pentose phosphate pathway.** Glucose-6-phosphate (G6P) has several fates in glycolysis (Glyc), glycogen synthesis, and ribose synthesis via the pentose phosphate pathway (PPP). UDPGlc = uridinediphosphoglucose; R5P = ribose-5-phosphate; nt = nucleotide. Enzymes in blue: PYG: glycogen phosphorylase; GYS: glycogen synthase; UGP: UDP-glucose pyrophosphorylase; PGM: phosphoglucomutase.

Analogous to protein synthesis, nucleic acid synthesis can be determined by tracing the pathway from de novo nucleotide synthesis into the end products RNA and DNA. The nucleotide pathways are conveniently probed using a combination of ^13^C and ^15^N sources, predominantly glucose and glutamine. Glucose via G6P or F6P usually provides the carbon for the ribose subunit via the PPP, leading to PRPP (Figure 4).

Nucleobase synthesis then flows through two separate pathways: purines via the pathway that builds directly onto PRPP using glycine, bicarbonate, methylene tetrahydrofolate (carbon), and Gln and Asp (nitrogen), and pyrimidines via the pyrimidine pathway using bicarbonate and aspartate, followed by condensation of Uracil with PRPP [213]. These pathways can be determined by NMR and mass spectrometry and, with appropriate use of different tracers, distinguish which sources contribute most [19,111,214]. Determining the incorporation of labeled nucleotides into polymeric nucleic acids can be achieved by digestion to mononucleotides, followed by NMR or MS analysis [111].

These procedures can help determine the relative importance of de novo synthesis versus salvage pathways. When provided with high levels of glucose in standard media, cells in culture usually turn over the nucleotide pool using primarily exogenous glucose for ribose production via the PPP [98,215]. Under the same conditions, exogenous Gln generally provides more carbon than glucose to pyrimidine biosynthesis [111], and the total carbon labels for the pyrimidine rings and ribose are comparable [111]. In contrast, although de novo purine nucleotide synthesis is substantial in rapidly dividing cancer cells in culture, the amount of glucose-derived carbon found in the purine rings is considerably lower than either the Gln-derived nitrogen or the glucose-derived carbon in the ribose rings [19,65,110,216]. This suggests that, at least in these cases, exogenous serine and/or glycine (and thence the 1-carbon pools) effectively competes for glucose-derived Ser/Gly carbon [19], and the salvage pathways via recovery of bases from nucleotide catabolism contribute relatively little, unlike other tissues [217]. In other systems, however, the salvage pathways may dominate [213,216,217,218]. Only multiple labeling approaches can really assess the sources of C and N for the de novo pathways versus the salvage pathways.

#### 2.5.3. Glycogen Turnover

Glycogen is primarily a polymer of glucose, though some other sugars such as glucosamine can be found in certain circumstances [219]. Glycogen may act as a storage reservoir of glucose and is typically abundant in the liver, skeletal muscle, kidneys, and brain [6,219,220,221,222], but is also found in other organs including tumors [223,224]. It can be analyzed by digestion to glucose, either enzymatically [219] or by acid hydrolysis with focused microwaves, or intact by NMR [224].

Glycogen is synthesized by successive addition of activated glucose (via UDP-glucose), mainly in α-1,4 linkages, with occasional (ca. 10 residues) α-1,6 linkages, giving the highly branched and relatively compact structure of glycogen. The activation of glucose for synthesis is a net consumer of metabolic energy (UDP is converted to UTP by phosphorylation with ATP). Glycogen is successively broken down by phosphorolysis, i.e., using inorganic phosphate to produce Glucose-1-phosphate (catalyzed by one of the tissue-specific glycogen phosphorylase isoenzymes). This product can then enter glycolysis after isomerization to G6P (Figure 4). As glycogen-derived G6P does not consume an ATP (unlike the hexokinase reaction), the net ATP yield of glycogenolysis plus glycolysis is three ATP/glucose [225]; the glycogen storage prepays one of the ATP-consuming steps. Clearly, the metabolism of glycogen can be very important in the energy homeostasis of cells under different conditions.

As the net synthesis of glycogen utilizes UDP-glucose, the supporting pathway includes the conversion of glucose or glucose-phosphate to G1P and thence to UDP-glucose. G6P is thus a critical hub for glycogen synthesis and degradation, as well as glycolysis and the pentose phosphate pathway. All of the relevant intermediates can be measured in detail using ^13^C tracers such as [U-^13^C]-glucose or ^13^C_5_ Gln as all of the intermediates can be quantified by MS + NMR, as well as the pyrimidine synthesis pathway [224].

Much of metabolism and metabolomics is concerned with measuring or tracing “small” molecules, operationally defined at Mr < 1000–1500. As discussed here, quantitatively, it is the anabolites that account for most CHONPS supplied to the cells or tissue. Yet tracer balances are rarely if ever recorded, unlike the older classical radiotracer studies [226], as stable isotope incorporation into macromolecules is challenging. Even estimates of glucose conversion to lactate can be difficult to reconcile with proliferation [108]. However, tracer atoms entering different components of these major sinks can be very informative about the overall nutrient utilization for cell proliferation, for example.

## 3. Complex Metabolic Models

### 3.1. Two-Dimensional Cultures

Although two-dimensional (2D) cell culture has many advantages in terms of experimental control of conditions and the ability to manipulate gene and protein expression in the entire population, even if realistic growth media and oxygenation levels are used, the cells are not homogeneous, and their morphologies are usually quite different from that in the tissue of origin. It is well known that transcriptional profiles, and, perhaps even more so, metabolic activity, is very sensitive to these conditions [106,227], such that cells grown in 2D culture do not closely resemble the behavior in more complex systems [19,86,228]. Notably, 2D cultures rather poorly predict responses to drugs, for example [43,229,230,231]. Nevertheless, these models are still widely used as they have the most options for experimental control of conditions, genetic manipulations, and comparative simplicity for modeling purposes. For these reasons, 2D cell cultures are widely used for SIRM studies [17,21,101,187,232].

Although genetic manipulations by CRISPR/Cas9 [233,234], siRNA, shRNA [235,236], and antisense oligonucleotides [237,238,239] are fully compatible with 2D cultures, more complex systems are less amenable to these kinds of manipulations.

### 3.2. Three-Dimensional Spheroids/Organoid Cultures

Given the many limitations of 2D cell cultures, increasing effort is being placed in 3D cultures and organoids [240,241], which have been available for many years [231,240,242,243,244,245,246,247,248,249,250].

There are numerous ways to promote 3D culture formation with various supporting matrices [44,244,247], induced aggregation by magnetic fields after uptake of magnetic nanocrystals [250], in hanging drops [250], or growth on very-low-retention surface wells [248,251]. Matrigel and similar support matrices have several disadvantages for metabolic studies, not least the variable composition of Matrigel and difficulty in digestion to release the spheroids rapidly [44]. Magnetic particles are optically opaque, so the spheroids cannot be monitored or stained for microscopy/IF [245]. Interestingly, at least some human cancer cells produce their own ECM when cultured as spheroids [228], which is presumably more relevant than the variable ECM extracted from mouse sarcoma [252].

As the spheroids are relatively small, and can be easily grown in standard plates, there is complete control over the medium composition including the addition of inhibitors and tracers, as for 2D culture. Depending on the cell type, it may be possible to create spheroids from multiple cell types, further mimicking the natural environment [253], which, in tissues, comprises multiple cell types (see below). In addition, to discriminate between the effects of direct cell–cell interactions and those via diffusible compounds released from one cell type, the co-culture can be compared with a culture in which two cell types are separated by a membrane that allows free diffusion of small molecules but not exchange of cells. As with 2D cell culture, subsampling of the medium is possible to determine uptake rates of particular nutrients and excretion of metabolites by analyzing the media at timed intervals.

For cells that can divide (i.e., have not reached the Hayflick limit or become senescent [254]), genetic manipulation of the cells can be readily carried out in the 2D state, which are selected and induced to form spheroids, which of course may differ morphologically from the parent cell and/or have altered metabolic requirements.

In order to induce stable spheroid culture, it is normal to use media that promote stemness, which include various growth factors and sometimes very high concentrations of compounds such as nicotinamide [255]. In this sense, the control over the medium is not as free as for 2D cell culture, and has to considered in the design phase.

Seahorse analysis on spheroids grown from colorectal or pancreatic cancer cell lines in the absence of matrices behaved quite differently from the 2D cultures, associated with changes in the balance between lactic fermentation and coupled mitochondrial respiration, correlating with altered expression of the monocarboxylate transporter and the mitochondrial protein translocase TOMM20 [256].

Sato et al. [257] used metabolomics to study spheroids formed from OVTOKO (ovarian clear cell adenocarcinoma) and SiHa (cervical squamous cell carcinoma) cell lines, in comparison with the profiles obtained in 2D culture. PCA analysis showed separation between 2D and 3D cell cultures and especially increased levels of Ser and Gln, as well as adenylates.

Similarly, our group compared 2D and 3D cultures of A549 (NSCLC) and pancreatic adenocarcinoma (PANC1) cells using magnetic beads, with SIRM analysis with [U-^13^C]-glucose [245]. Although the extent of ^13^C incorporation into metabolites of glycolysis, the Krebs cycle, the pentose phosphate pathway, and purine/pyrimidine nucleotide synthesis was largely comparable between 2D and 3D culture systems for both cell lines, the 3D cultures showed reduced capacity for de novo synthesis of pyrimidine and sugar nucleotides. More strikingly, selenite induced much less perturbation of these pathways in the spheroids relative to the 2D counterparts in both cell lines, which is consistent with the corresponding lesser effects on morphology and growth, and shows an impact of culture on response to toxic agents. SIRM analysis of mixed cell spheroids (co-cultures) also show metabolic differences compared with the pure cell versions or 2D cultures, which has implications for immune responses in terms of the tumor microenvironment [44]. To date, there have been few studies of drug response analyses in spheroids using tracer-based metabolic readouts.

### 3.3. Organotypic Cultures

The next level of complexity above organoid cultures is tissue slices. Thin sections of tissue can be incubated in controlled media, enabling the influence of nutrient supply or inhibitors to be determined using tracers as for 3D culture [228,258]. The tissues can be freshly resected from patients or from cryopreserved tissues and incubated for long periods (e.g., one month, depending on the organ of origin) [259]. The advantage of these organotypic cultures is that they represent individual subjects, the cells have not been cultured over several generations under unphysiological conditions (which changes the expression profile [260,261,262]), and they retain all of the original cell types and tissue architecture, without being connected to the circulatory system as in vivo studies (see below) [44,263]. As such, they recapitulate the biochemistry and response to drugs more accurately than other models [55,264]. Further, compared with mouse xenografts, this model often has non-cancerous tissue from the same organ available that can be used as a matched control. In the mouse xenograft model, the control tissue is of mouse origin, not human, and is thus not paired.

Warburg [133] first demonstrated accelerated lactic fermentation in the presence of oxygen using thin tumor tissue slices placed on moist filter paper. He showed that the slice thickness needed to be ca. 0.5 mm to avoid significant diffusion limitation [133]. Most tissues are not exposed to air on one side. Two-sided diffusion is commensurate with minimizing diffusion barriers even at 0.7 mm, provided that the tissue is bathed in a medium that also moves with respect to the tissue, which also dilutes waste products [264]. Much thinner slices can be very fragile, and the relative proportion of damaged to undamaged cells increases as slice thickness is decreased.

**Figure 5:**
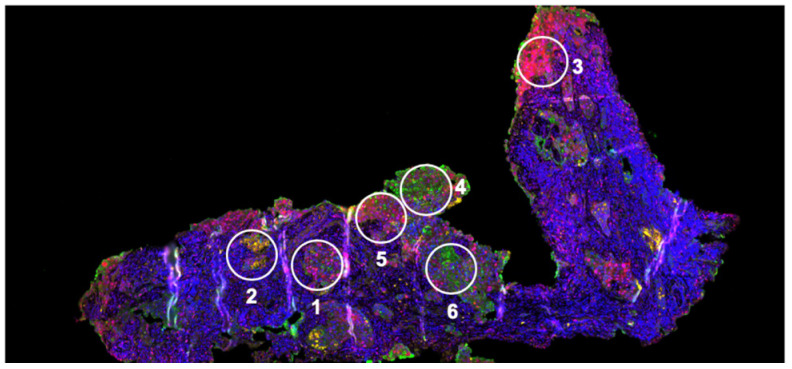
**Tissue heterogeneity in lung cancer tissue**. This slice (10 μm thickness) was stained for cancer (panCK, green), CD8- (T cells, red), and CD68- (macrophages, yellow) expressing cells and DAPI (localizing nuclei, blue). Different regions (circled) were analyzed by transcriptomics, whole slices by SIRM using ^2^H Glucose + ^13^C,^15^N Gln and analyzed by NMR and ultra-high-resolution mass spectrometry. The circled regions of interest (100 μm diameter, numbered 1–6) were further analyzed for the protein expression level of 58 different markers of cancer and immune cell functional states using oligonucleotide-barcoded antibodies using the NanoString Digital Spatial Profiling system. From Figure 4C of Fan et al. [44] under creative commons https://creativecommons.org/licenses/by/4.0/ (accessed on 1 May 2024).

Keeping track of the slices within a tumor actually provides limited spatial resolution through the tissue (i.e., in one dimension) as each slice can be independently analyzed. Thin tissue slices are also commensurate with (immune)histochemical staining and microscopy (Figure 5), as well as transcriptomic analyses either at regional levels [44] or at essentially single-cell resolution [265,266]. As the tissue slices retain all of the original cell types, metabolic readout of the entire slice represents an average, such that contributions from different cell types or the impact of interactions between cells can be difficult to disentangle. Spatially resolved transcriptomics and protein analyses can help deconvolute this layer of complexity [265,266]. In addition, the metabolic phenotypes of individual cells derived from the tissue, and knowledge of cell-specific metabolism, can also be used to assess cell-dependent metabolic phenotypes and response to drugs or altered conditions [44,98].

Although generally less efficient than in 2D cell cultures, methods for manipulating gene expression by selective uptake of siRNA or shRNAs [267] are being developed which can complement studies on small-molecule inhibitors. Using Accell RNA, Ruigrok et al. [268] reported successful transfection of 250–350 μm thick mouse lung and kidney tissue slices [268]. Further, mRNA delivery using mRNA-based antiviral approaches [269] might be developed to produce any protein of interest in target cells.

Such organotypic tissue cultures have been used for metabolic studies and assessing the tissue microenvironment and heterogeneity in several systems, including lung cancers [90,98], liver, kidney [268,270,271], brain [272], breast [259], and prostate cancer [273].

### 3.4. Organ Cultures

Perfused organ cultures such as the Langendorrf perfused heart [274,275,276], kidney [277], liver [278,279,280], everted intestine [281], and skeletal muscle [282] have long been used in physiological studies. These models have the advantage of representing the entire organ in its full functional complexity while being able to control the supply of labeled nutrients and the removal of waste products. Generally, the biochemistry of the cells is better maintained in these systems (for example, hepatocytes are notoriously difficult to culture [232,283]). Usually, such studies are carried out with organs excised from experimental animal models.

Organ cultures are amenable to MRI/MRS (see below), which gives an organ-level overview of energy metabolism when using ^31^P NMR and central metabolism when using ^13^C-enriched substrates. However, it is difficult to separate out the metabolic contributions from different cell types and, generally, after completion of the physiological experiments, the tissue can be extracted and subjected to high-resolution NMR and mass spectrometry to analyze otherwise poorly resolved or invisible metabolites due to immobilization or low abundance. The circulating medium can also be temporally subsampled to assess nutrient uptake and waste product release as for cell culture (see above), allowing a direct connection to the relevant physiological state(s).

### 3.5. Animal Models

In vivo studies in animal models have the advantage of a live organism, with all the disadvantages of the mixed cell and tissue types, plus the added complexity of intertissue communication via the humoral system, which can complicate metabolic analysis.

Tracer administration is usually systemic, which means that the isotopically enriched precursor reaches every tissue in the organism, which each have different uptake and metabolic rates. The different cells and tissues will then produce variable sets of labeled metabolites, some of which are released into the blood [284] and can be absorbed by distant organs. It is thus important to measure the isotopologue distributions in multiple organs and the blood to assess origins and exchange processes [285]. This becomes increasingly severe the longer the duration of labeling and can make achievement of an isotopic steady state difficult [91,286].

Tracer administration is an additional consideration, as there are numerous ethical and biological constraints that must be addressed. The main routes of isotopically enriched precursors are oral, either by oral gavage [95] or inclusion in water or feed [286]; by bolus injection directly into a vein [42,284] or intraperitoneally [38]; via continuous infusion into a vein [84]; or through implantation of an osmotic pump [285,287]. With the exception of ad libitum diet, all of these methods require animal handling with restraints or surgery and possibly with anesthesia, which impact metabolic rates either directly or indirectly.

Bolus injection via a vein introduces the desired tracer that is distributed systemically very rapidly [284] and thence to tissues, which have their own characteristic uptake and metabolic rates. The time course of humoral distribution and overall metabolism can be easily assessed by sampling blood and measuring the amount of labeled precursor present, as well as how much has been converted to metabolites that are excreted back into the blood. For example, using [U-^13^C]-glucose, we found that the optimal time to harvest organs was about 15 min post injection, when the lactate enrichment was at a maximum. In practice, we used three injections spaced at 15 min intervals to increase the degree of labeling of central metabolites. However, with a bolus injection, many of the more slowly turning-over pools (nucleotides, fats, proteins) were not highly labeled. By injecting glucose into the blood, much of it is actually metabolized to lactate by the red cells, which accounts for >40% of the blood volume. Furthermore, with a bolus injection, an isotopic steady state cannot be achieved, making modeling more involved.

Oral gavage is an alternative when absorption through the gut is desired and may lead to labeling of the gut microbiome [288]. Gavage can be stressful to the animal, and as it is also a bolus method, the time course of isotope distribution is complex and does not reach an isotopic steady state. Nevertheless, high levels of enrichment can be obtained in certain organs such as brain and liver [289].

For much longer labeling periods (e.g., >6 h), dietary administration of enriched precursors may be more appropriate. Ad libitum feeding has the advantage of no added stress to the animal or requires anesthesia, but is complicated by animal feeding behavior and shows a complicated time dependence. A liquid diet that is formulated for either glucose or Gln tracer administration is a convenient way to label slowly turning-over metabolite pools in rodents. The commercially available diets are adjusted to maintain the original levels of carbohydrate and nitrogen. For mice, which eat mainly nocturnally, 18 h of feeding during the dark cycle is sufficient to observe label incorporation into slowly changing macromolecular metabolic pools including RNA, complex lipids, and proteins [286].

Implanted osmotic pumps are widely used for drug delivery in animal models [287] and offer an additional means for continuous infusion that can supply labeled precursors at a defined rate over an extended time period (e.g., 24 h), and after recovery from surgery, they do not rely on ad libitum feeding [285]. In an early application, Xu et al. [285] used a subcutaneous minipump to infuse fasted mice with ^13^C_6_-glucose, ^13^C_3_ lactate, or ^13^C-2 lactate over 24 h. Isotopologue distribution analysis of blood plasma enabled hepatic glucose production (HGP) and glucose carbon recycling lactate and glycerol turnover at the whole organism level, using a physiological organ model. Very-long-term constant infusion may not mimic natural physiology, and implantation, depending on the site, may introduce adverse effects if a major organ is also catheterized [290].

As animals need constant access to water, labeling precursors can be incorporated into their water supply [291], including deuterated water. This route has been widely used for administering drugs and heavy water [292] as well as in humans as the glucose tolerance test and ^13^C imaging spectroscopy [293]. Although animal studies can be complex, the experimental design should seek to maximize information retrieval relevant to the questions being posed, rather than for the convenience of the researchers.

### 3.6. Human Subjects

Major differences between small animal models and human subjects are size, additional ethical and legal regulations [294], and far greater intrinsic variance due to both genetic and environmental considerations. The size difference (20 g mouse versus 70+ kg human) means a very different metabolic rate (approx. seven-fold) that needs to be accounted for, as well as the quantity of labeled precursors that is needed to achieve a measurable amount of labeling. The routes and methods of administration, however, are the same as for other mammals, with similar considerations for experimental design in the context of the question(s) to be answered [55,295].

For example, bolus injection of ^13^C_6_ glucose into human subjects requires around 10 g of pure, sterile glucose, with blood sampling and tissue collection within 2–3 h of injection. This approach showed the activity of pyruvate carboxylase, which was overexpressed in NSCLC tissue [34,37], which also indicated that PFK1 activity was not rate-limiting for glycolysis in the tumors. Hensley et al. [62] demonstrated the metabolic heterogeneity in NSCLC by continuous infusion of ^3^C_6_ glucose, and that lactate can act as a nutrient in some regions of the mass. These studies also demonstrated that accelerated lactic fermentation was present in the tumors compared to the adjacent matched non-cancerous lung tissue (an example of the Warburg effect in situ). Similarly, ^13^C_6_ glucose infusion into very young children bearing neuroblastomas showed substantial tumor mitochondrial activity of both PDH and PC, as well as high lactate labeling and production of catecholamines [41].

Brain metabolism has been extensively studied using glucose infusion by ^13^C MRS, which showed activity of the Krebs cycle, and by modeling the interaction between neurons and glia (Gln cycle) [67,296].

Heavy water infusions have been used to characterize protein turnover [297] that required daily does over a 2-week period and frequently for lipid metabolism in human subjects [298,299,300].

These studies further demonstrate the overall safety of stable isotope administration, as well as the ability to map specific metabolic activities in target tissues.

## 4. Spatially Resolved Metabolism and Models

Spatial resolution can be at the organ level, sub-organ tissue level, single-cell level, or, ultimately, at the subcellular level [15,44,301,302,303,304,305,306]. The biological question at hand largely determines the spatial resolution needed, though technological issues will always limit what is possible. Spatially resolved analyses can be achieved by dissection, for example, intact organs even down to small groups of cells (cf. laser microdissection [307]); see above. More generally, however, spatially resolved studies involve imaging.

### 4.1. Amount versus Volume. Sensitivity: Imaging In Situ versus Dissociation

As the spatial resolution increases, the target size decreases quadratically for thin tissue slices or decreases cubically in volume. This means that the sensitivity of the detection may become limiting at very high resolution. The tradeoffs are therefore among target size (volume), concentration of analytes, acquisition time, and depth of metabolic coverage. Point-by-point rastering is the slowest modality, and imaging time is determined by the number of points sampled and the dwell time at each pixel. High resolution with adequate sampling over a wide field can therefore be very time-consuming (hours per sample).

Human cells range in volume from <0.5 pL to 1–2 pL (epithelial cells) to 5 pL (liver cells). A diploid nucleus occupies ca. 20–25% of the total volume [308], and mitochondria may occupy 10–30% of the total volume depending on the cell type and state [309], so that the cytoplasmic volume may be of the order 50% of the total volume.

Table 2 shows the amounts of metabolite present at different concentrations, for a sampling of a large group of cells (10^9^) down to individual cells. For a large number of cells, even low-abundance metabolites present at nM concentrations would be detectable and quantifiable by mass spectrometry, whereas at the single-cell level, even a 10 μM concentration becomes challenging (amole range), especially with complex biological samples where background chemical noise will contribute significantly to the overall signal. For subcellular probing, the challenge is even greater. This ultimately limits the metabolic coverage attainable with even the most sensitive detection system.

There are important metabolites present at high concentrations (>100 μM) in cells, including ATP, NAD^+^, GSH, most amino acids, and some organic acids, but many are present at much lower steady-state concentrations, and signaling molecules are typically sub nM.

### 4.2. Intracellular versus Extracellular Pools

The extracellular space is filled with a wide variety of macromolecules and small metabolites that form a complex microenvironment called the extracellular matrix (ECM). This space also contains the underlying solution that is isotonic with cells and is fundamentally similar to plasma. A wide range of metabolites are present in this interstitial fluid [145,310], which cells may exploit as a source of nutrients via various mechanisms [145,146,152,153,172,311,312]. With point-by-point imaging techniques, the spot size may include extracellular as well as intracellular metabolites, thus smearing the true biochemical and spatial resolution. Nevertheless, spatial resolution even at the meso scale is very important for tissue analysis, owing to the high degrees of cellular and structural heterogeneity, especially under pathological conditions. Figure 5 shows a stained section of slices of NSCLC and non-cancerous lung that shows the extreme variation in positional distribution of different cell types, which likely interact both via direct contact and via diffusible metabolites.

### 4.3. Metabolic Imaging by MRI/MRS

NMR in MRI/MRS has long been used for anatomical and metabolic imaging [76,313,314,315,316,317,318,319,320], which, because of the low energies involved, are compatible with live organism studies utilizing stable isotope tracers such as ^13^C, ^2^H, and ^31^P (which is 100% naturally abundant). With modern imaging spectrometers, large-scale (wide-field) anatomical imaging is possible relatively quickly as the image is generated as a planar slice [321], multiple slices can be recorded simultaneously with an array of detector coils (parallel imaging) [322], and the depth of field is commensurate with whole-body imaging and spectroscopy.

The spatial resolution of MRS is typically low (at best several mm^3^) owing to the low intrinsic sensitivity of NMR (rule of thumb requires 1 nmol for high-resolution spectroscopy and considerably more for imaging-based spectroscopy). Again related to the intrinsic sensitivity of NMR detection, coupled with moderate spectral resolution, the metabolite coverage is relatively low in MRS, limited to a few (key) abundant metabolites [76,320,323]. Nevertheless, using a variety of stable isotope-based studies of patients and organisms has provided gold-standard information about metabolism in live tissue in situ in the brain [67,75,77,84,324,325] and liver [326], among others [327]. More recent developments in dynamic nuclear polarization (DNP) have greatly increased the sensitivity at the cost of low metabolic coverage, and short half-life of the hyperpolarized spins [78,79,80,81,328,329]. However, as the measurements are made in vivo, critical dynamic information can be obtained about central metabolic pathways related to pathologies [80,329,330]. Advances in detection efficiency, such as indirect detection of ^13^C by the attached proton [331,332], and advanced data reduction techniques [320] should permit large gains in sensitivity in SIRM studies in vivo that benefit from signal averaging and much longer acquisition periods than is possible with DNP, at the cost of decreased temporal resolution.

### 4.4. Confocal Microscopy

The spatial resolution of confocal fluorescence microscopy can be very high (<1 μm), with typically low metabolic coverage owing to the poor spectral resolution of fluorescence and the need for specific probes which can be complicated by uptake kinetics in tissues. However, optical methods also have low penetration depth, so are usually limited to thin sections. With optical tissue clearing, greater depth can be achieved, albeit only in vitro [333]. The imaging time using point-by-point microscopy can be lengthy, so very good environmental control is needed for live tissue imaging. The imaging times can be reduced using spinning disk confocal microscopy (essentially linear detection) or maximally using light sheet microscopy with CCD camera detection (planar imaging) [334,335].

In general, the metabolic coverage by fluorescence confocal imaging is limited to a few intrinsically fluorescent species (e.g., flavins, NAD(P)H, fluorescent sensors such as TMRE [336,337]) or to fluorescently tagged precursors such as NBDG glucose [336,337] and BODIPY-labeled fatty acids [338]. An increasing number of fluorescence-labeled metabolites are becoming available, which should substantially enhance the metabolic coverage of optical microscopy [339,340,341].

Redox ratios can be monitored at subcellular resolution using fluorescent sensors, which have to be introduced into cells [342]. As the proteins can be tagged, they can be made to localize to different compartments [343], showing different redox ratios in the cytoplasm versus the mitochondria in live cells, for example [344]. The biosensors probe the free (i.e., unbound) metabolites, and if both oxidized and reduced forms of NAD(P):NAD(P)H can be measured, the true redox ratio can be determined, in principle in real time and compartmentally localized. For example, although the total dinucleotide concentration is of the order 0.5 mM in mammalian cells, the concentrations and redox ratios differ significantly different between the cytoplasm and the mitochondria. Further, the free concentrations are much lower than the total amounts, owing to binding to the large number of dehydrogenases present [15]. This also leads to NAD^+^/NADH and NADPH/NADP^+^ ratios quite different from the ratios of the total concentrations. The cytoplasmic ratio of free NAD^+^ to NADH is 500–1500 [155,344] compared with <10 in mitochondria [345]. In contrast, the free NADPH/NADP^+^ ratios are in the range of 20–80 (cytoplasm) and ca. two-fold higher in mitochondria [343].

The advantage of optical confocal microscopy is the excellent spatial resolution and compatibility with live cell analysis, but its low metabolic coverage and insensitivity to isotopic substitution is a significant limitation. In principle, confocal vibrational spectroscopy can combine the spatial resolution advantages of optical methods, with much greater chemical diversity, as well as being sensitive to isotopic substitution. Replacing C-H with C-D causes a large change in vibrational frequency [346], and ^13^C/^15^N substitutions also give rise to detectable isotope shifts [347] and could be used to follow biochemical transformations, including NAD(P)H changes. Both Raman spectroscopy and infrared (e.g., via photothermal excitation) have been used to determine a wide range of metabolites in cells and thin tissue sections at subcellular resolution [346,347,348,349,350].

### 4.5. Single-Cell Analysis by MS

The high intrinsic sensitivity of mass spectrometry makes detection of sub fmol levels of metabolites possible, and therefore enables metabolomics of common relatively abundant metabolites in individual cells [15,306,351,352,353] or even organelles within cells [306]. While tissue dissociation into individual cells loses the biologically important spatial information, this can be retained by ablating individual cells using MALDI-based mass spectrometry imaging [354,355,356] or DESI-MS [357,358,359,360,361,362,363,364], or groups of cells isolated by laser capture microdissection coupled to MS detection [362,363].

Abundant metabolites can be mapped, including lipids [362,364,365], glycogen [366], and glycosylation of proteins [219,367]. Most of these studies have been at the level of groups of cells and steady-state levels, limited by spot/beam size, matrix, and sensitivity [365,366,367,368] (see above). MALDI requires adding a matrix to the tissue surface, which introduces unwanted ions and may interfere with biochemistry. As thin slices are needed, imaging time to obtain depth can be very long, even at modest x-y spatial resolution. DESI does not require a matrix or a vacuum as it uses a stream of charged molecules for desorption [368,369,370,371]. Typically, MALDI spot sizes are around 30–50 μm, though smaller sizes have been reported. Similarly, DESI may work at an effective resolution of 50 μm, though newer developments are increasing resolution to the 10–20 μm level, which is essentially the single-cell level. A variant of DESI-MS called AFADESI (air-flow-assisted desorption electrospray ionization) showed good coverage in brain chemistry [357]. Nano-DESI is a development that improves the sampling from tissues using continuous flow, which can be interfaced to a variety of separation modes prior to MS with resolutions down to 12 um [372,373]. Applications of MALDI and DESI-based imaging to several cancers has been reviewed, showing the use of a small number of assigned ions to discriminate between cancerous and non-cancerous tissue [357].

Carson et al. [374] used DESI-MS to map lipid turnover in a mouse brain following feeding 8% deuterated water over a period up to 40 d, at a (pixel) resolution of 75 × 150 μm, by which time the deuteration had still not reached a steady state (but was well represented with simple kinetic curves), showing that certain large pools of metabolites very slowly turn over compared to intermediates of central metabolism. The time-dependent maps showed distinct differences in specific lipid distributions in various parts of the brain. More recently, ^13^C labeling has been incorporated to provide information about intracellular metabolites at the tissue level [304,358]. SIRM approaches have been applied to brain tissue slices with detailed spatial resolution showing increased glucose metabolism via glycolysis and the pentose phosphate pathway [375]. Hu et al. proposed an algorithm for single-cell spatial resolution in tissues with protein markers of the cells to enhance cell metabolite assignment [376].

MALDI and DESI MS imaging have been combined with ^13^C glucose labeling of gliomas in mice, using a liquid diet [286] to administer the tracer over 24 h. Thin tissue slices of the brain were imaged at a resolution of 50 μm, and the isotopologue distributions in several abundant metabolites of central metabolism were determined, including estimating fatty acid synthesis rates (flux). The estimates of pool sizes from MALDI and DESI, however, were shown to be inconsistent, which indicates that caution is needed when comparing metabolite abundances across multiple images [374].

As Table 2 indicates, single cells contain sub attomole amounts of metabolites present at sub μM concentrations. Individual mitochondria have a volume of ca. 1 fL, so even at a concentration of 1 mM, the amount of a metabolite is 1 amole. At such trace levels, not only is detection by mass spectrometry difficult, even assuming essentially 100% ionization efficiency, but contamination from background species becomes increasingly problematic, and more controls of blanks have to be recorded. Tracers may help overcome contamination as only active metabolites can become labeled, provided there are no trace contaminating organisms also present.

### 4.6. Future Directions

Although high-resolution imaging modalities with subcellular resolution are currently possible, the metabolic coverage of optical detection is very limited, restricting studies to very well-posed metabolic questions. Such studies can be carried out in live cells or thin tissue slices, thus providing both spatial and temporal readouts of the biochemistry. However, fluorescent probes are insensitive to isotopic substitution by metabolic processes, also limiting the biochemical depth of studies. Optical methods also have penetration depth issues, and although tissue clarification goes a long way in circumventing that limitation, it probably interferes with the actual metabolic profile and may not be fully compatible with in vivo analyses. The metabolic coverage will, however, continue to increase as more probes of metabolic processes become available.

Vibrational spectroscopy imaging has a much greater metabolic coverage, at the expense of very complicated spectra which can make deconvolution problematic. Simplifications can be achieved using SERS [377] and in some cases resonance Raman enhancements [378]. Furthermore, vibrational spectroscopy is sensitive to isotopic substitution and compatible with live cell imaging. Again, however, the penetration depth is problematic for organ-level metabolic analyses. The low sensitivity of Raman, for example, also requires very long acquisition times for a modest field of view and is therefore not compatible with broad-range temporally resolved metabolism.

Mass spectrometry has by far the widest metabolic coverage and is sensitive to isotopic substitution, though for macromolecules, this can lead to such a large number of isotopologues that the spectra become uninterpretable. MS imaging also has very low depth penetration and is not generally compatible with live tissue or organs, though in principle, DESI could be compatible with live tissue. A promising development using MS, however, is intraoperative surgical analysis of tumors, using pyrolysis–MS in real time; the so-called iKnife has been shown to be able to distinguish between cancerous and non-cancerous tissue with good accuracy and thus may help guide marginal resections [379,380].

Nevertheless, the achievement of subcellular spatial resolution of a substantial number of metabolites continues to need development. The simultaneous use of stable isotope labeling exacerbates the sensitivity problem, as well as spectral overlap, as many more species are produced. Lipids, for example, may have a very large number of isotopologues that all have to be quantified to determine fractional enrichment [20]. Furthermore, the act of tissue preparation (slicing, mounting, etc.) may produce smearing artefacts by physical forces and/or by diffusion. Although diffusion is slow, small molecules may diffuse hundreds of μm in an hour in unfixed tissue without a cryostage [381].

The very large quantities of data that are generated by omics require powerful and sophisticated software, especially where different omics data streams (i.e., transcriptomics, proteomics, and metabolomics) are to be integrated. Given the increasing power of machine learning/AI tools, this would likely be a rich area for the application of such tools [369,382,383].

## 5. Conclusions

There are no methods that can currently meet the need to obtain deep metabolic coverage with high (subcellular) spatial resolution in live tissue or organisms. This necessitates workarounds that are geared to answering specific questions. However, the spatial (and temporal) resolution and metabolic coverage needed depend on the problem posed, and often modest (regional) spatial resolution or highly targeted metabolite determination can suffice. Further, considerable progress is being made in metabolic imaging of tissue slices ex vivo with stable isotope tracers, and as both sample handling and analytical technologies advance, more metabolic depth should be achieved at the desired spatial resolution, enabling detailed metabolic network dynamics to be extracted.

## Figures and Tables

**Table 1:** Common stable isotope-enriched precursors.

Compound	Isotopomer	Metabolic Pathways	Refs
Glucose	^13^C_6_	Glycolysis, glycogen synthesis, Krebs cycle, HBP Lipid synthesis	[36,67,68] [20]
	^13^C-1	Glycolysis	[69]
	^13^C-2		[70]
	^13^C-1,2	Pentose phosphate pathway;	[71,72,73]
		PC/PDH	[63,64]
	^13^C-3,4	Pentose phosphate pathway	[74]
	^2^H_7_		[75]
	^2^H-6,6		[74,76]
Fructose	^13^C_6_	Fructose metabolism	[60]
Lactate	^13^C_3_	Lactate utilization, gluconeogenesis	[35,77]
Pyruvate	^13^C-1	LDH activity (via DNP)	[78,79,80]
	^13^C-2	DNP-LDH, Krebs cycle activity	[81]
Acetate	^13^C_2_	Krebs cycle	[82,83,84]
Glutamine	^13^C_5_	Glutaminolysis, Krebs cycle, protein synthesis, gluconeogenesis	[85]
	^13^C_5_^15^N_2_	Glutaminolysis, Krebs cycle, transamination, amidotransferase, gluconeogenesis, nucleobase synthesis	[18,85,86,87]
	^13^C-1; ^13^C-5	Reductive carboxylation	[88]
	^15^N-2, ^15^N-5	Amino and amido transferase activity	[89]
Tryptophan	^15^N_2,_ [U-^13^C]	Protein, kynurenine/nicotinamide	[90,91]
V,I,L	^13^C,^15^N	Branched chain amino acid metabolism	[92]
Glycine	^13^C_2_	Protein, purines, 1-C metabolism	[19,93]
Serine	^2^H_3_	Phospholipids, protein, 1-C metabolism	[19]
Methionine	ε-^13^C	1-carbon metabolism via S-Adenosylmethionine	[94]
Palmitate Oleate	^13^C_16_ ^13^C_18_	Fatty acid uptake and metabolism	[95,96,97]
Water	^2^H_2_O	Lipid synthesis	[59]

**Table 2:** Relationship between volume, concentration, and quantity.

No. Cells	Total Cell Volume ^a^ nL	N Mole @ 1 mM ^b^	N Mole @ 10 μM ^b^	N Mole @ 0.1 μM ^b^	N Mole @ 0.001 μM ^b^
1E9	2E6	2E-6	2E-8	2E-10	2E-12
1E6	2000	2E-9	2E-11	2E-13	2E-14
1E3	2	2E-13	2E-14	2E-16	2E-18
1	0.002	2E-15	2E-17	2E-19	2E-21

^a^ assuming 2 pL/cell; ^b^ averaged over the entire cell volume.

## Data Availability

No new data were created or analyzed in this study. Data sharing is not applicable to this article.
